# Examining associations between physical activity context and children meeting daily physical activity guidelines: the role of outdoor play, sports, and other organized activities

**DOI:** 10.3389/fpubh.2024.1352644

**Published:** 2024-05-20

**Authors:** Christopher D. Pfledderer, Denver M. Y. Brown, Nalini Ranjit, Andrew E. Springer, Raja I. Malkani, Deborah Salvo, Deanna M. Hoelscher

**Affiliations:** ^1^Department of Health Promotion and Behavioral Sciences, Michael & Susan Dell Center for Healthy Living, University of Texas Health Science Center Houston (UTHealth Houston) School of Public Health in Austin, Austin, TX, United States; ^2^Department of Psychology, The University of Texas at San Antonio, San Antonio, TX, United States; ^3^Michael & Susan Dell Center for Healthy Living, University of Texas Health Science Center Houston School of Public Health in Austin, Austin, TX, United States; ^4^Department of Kinesiology and Health Education, College of Education, The University of Texas at Austin, Austin, TX, United States

**Keywords:** physical activity, context, children, sports, outdoor play, organized activities, active travel

## Abstract

**Background:**

Less than one-quarter of US children meet physical activity (PA) guidelines. Understanding the context in which PA occurs and how these contexts may play a role in meeting PA guidelines is an essential step toward developing effective behavioral interventions. The purpose of this study was to examine associations between PA context (sports participation, participation in other organized physical activities, active travel to school, and outdoor play) and the number of days children met PA guidelines in a representative sample of children living in Texas.

**Methods:**

We analyzed cross-sectional data from a statewide sample of fourth-grade children in Texas who completed the 2019–2020 Texas School Physical Activity and Nutrition (Texas SPAN) survey. The Texas SPAN survey was designed to monitor the statewide prevalence of overweight/obesity among school children and assess habitual self-reported obesity-related behaviors, including diet and PA. Weighted Poisson regression models were employed to examine the associations between PA contexts (sports participation, participation in other organized physical activities, active travel to school, and outdoor play) and the number of days children met PA guidelines, adjusting for sex, race/ethnicity, overweight/obesity, urban–rural status, and economic disadvantage.

**Results:**

A total of 16.7% of fourth-grade children met physical activity guidelines every day during the week (mean age = 9.4 ± 0.6 years; female = 48.7, 51.8% Hispanic, mean days meeting PA guideline = 3.6 ± 2.3 days). One in ten (11.2%) children did not meet daily PA guidelines on any day of the week, and 72.1% met them between 1 and 6 days. Participating in sports (b = 0.22, 95%CI:0.14, 0.30), any other organized physical activities (b=0.13, 95%CI:0.017, 0.19), and playing outdoors 1–3 days (b = 0.25, 95%CI:0.04, 0.46) and 4–7 days in the past week (b = 0.77, 95%CI:0.57, 0.97) was significantly and positively associated with the number of days children met PA guidelines.

**Conclusion:**

Participating in sports, participating in other organized physical activities, and playing outdoors may beneficially influence the number of days children meet PA guidelines. PA programs should consider these contextual factors and investigate how to promote organized activities and outdoor play effectively and appropriately among children.

## Introduction

Physical activity (PA) is associated with many health outcomes in children including fitness and cardiovascular health (1), cognitive functioning, depression, and other mental health outcomes (2), and metabolic outcomes including overweight and obesity (3, 4). Current national guidelines from the American Heart Association and the Centers for Disease Control recommend children aged 6–17 years accrue at least 60 min of moderate-to-vigorous physical activity (MVPA) daily (5–8), yet based on current observational research, most children do not meet this daily recommendation (9). Furthermore, while there have been many high-quality PA interventions designed for and delivered to children, many with multiple components including buy-in from schools, families, and communities, few have been able to make a substantial impact on children’s PA (10–14). There is a need for more informative observational research to guide the development of these interventions in an effort to increase the proportion of children meeting PA guidelines in the US.

Children are exposed to a variety of opportunities (i.e., contexts) to accrue PA throughout their day, including physical education, recess, after-school programs, sports and other out-of-school structured activities, active travel, and unstructured free play at home and outdoors. These contexts differ in the degree to which they are structured, how they are made available to children, and their environmental and social correlates (10, 11, 13, 15–18). For example, sports participation is often delivered as a structured PA opportunity, overseen by adults at set times during the day/week, and often involves larger groups of children participating at one time (15, 16). There is also often a cost associated with sports participation, potentially limiting the opportunity for children from families who cannot afford it (19). Conversely, unstructured outdoor play (free play) is more likely done at recess or around the home environment, is rarely supervised, and often involves fewer children participating together at one time (20). While technically free from any associated monetary cost, playing outdoors may have other barriers including neighborhood safety and/or limited access to parks, recreation facilities, or play equipment at the home (20–23).

Understanding the differential influence of these contexts on children’s PA guideline adherence is key when designing maximally effective behavioral interventions targeting PA, as this could provide a way to identify potential intervention components that will have the best chance at “moving the needle.” There are many examples of PA-based interventions using different types of contexts as their mode of delivery including sports, after-school programs, and recess. Technically, differential success across these programs (10–13) should allow us to make some inferences regarding how different contexts may influence PA from these intervention studies. However, it is difficult to compare different contexts across studies due to unquantifiable heterogeneity. Additionally, we are not aware of any interventions that have been designed to compare how PA contexts may differentially impact PA outcomes in children (i.e., compare the effect of a sports intervention to a recess intervention). Thus, cross-sectional, observational data remain the only viable source of inference regarding these questions. Even though such data do not permit causal inference, it does provide the opportunity to compare PA behaviors across contexts with the same sample of participants in a naturalistic setting. It is also important to identify how differences in key demographics, like sex and socioeconomic status, play a role in PA context, as these may influence the dose of individual contexts children receive. For example, sports and other out-of-school structured activities are cost prohibitive, and certain PA contexts, like outdoor play and active travel, may be viewed by parents as more or less dangerous depending on the sex of the child, resulting in both socioeconomic and sex-based differences in PA context (23–27).

The Texas School Physical Activity and Nutrition (Texas SPAN) survey provides a unique opportunity to explore how different PA contexts associate with children meeting daily PA guidelines and to identify socioeconomic and sex-based differences in daily PA guideline adherence and differences in PA engagement across several contexts at a representative statewide level. While there have been previous studies exploring contextual factors associated with PA in children (17), and even some comparing PA engagement across contexts (28), most use small samples and have limited generalizability. Texas ranks 10th for rates of childhood obesity (29) and is home to nearly 7.5 million children, accounting for 10% of the entire US population of children (30), underscoring the need for more informative, large-scale, obesity-related behavioral research in this region. Therefore, the purpose of this study was to examine associations between PA contexts (sports participation and other out-of-school structured physical activities, active travel to school, and outdoor play), and the number of days fourth-grade children met PA guidelines in a representative sample of children living in Texas using data from the 2019–2020 Texas SPAN survey.

## Methods

### Study design

The Texas SPAN survey is a cross-sectional survey designed to monitor the statewide prevalence of children with overweight/obesity via objective measures of height and weight and assess habitual self-reported health-related behavioral outcomes, including diet and PA. It uses a stratified, multi-stage sampling plan to produce representative data for second-, fourth-, eighth-, and 11th-grade students in the state of Texas. The current study uses data collected from fourth graders during the 2019–2020 cycle of data collection. The Committee for the Protection of Human Subjects at the University of Texas Health Science Center at Houston (UTHealth Houston) (HSC-SPH-18-0432), the Texas Department of State Health Services Institutional Review Board, and local school district review committees reviewed and approved all study-related activities for Texas SPAN survey. Specific methodology is briefly described below, but detailed descriptions of the study have been reported elsewhere (31).

### Data collection and sampling

The Texas SPAN survey is a self-administered survey questionnaire administered to second-, fourth-, eighth-, and 11th-grade students in Texas. Survey items include questions about demographic characteristics, nutrition, PA, screen time, and dental habits. The survey has been previously tested for reliability and reproducibility (32). In addition to questionnaire items, Texas SPAN collects objective measures of height and weight used to calculate body mass index (BMI). Specific details on data collection methods have been reported elsewhere (31). Briefly, the Texas SPAN project is conducted, and data are collected by researchers at the Michael and Susan Dell Center for Healthy Living at the University of Texas Health Science Center Houston, School of Public Health in Austin. Data collection consists of survey administration and measurement of student’s height and weight to calculate BMI. All data collection procedures are completed in participating schools. A detailed process overview of all recruitment and data collection procedures has been published elsewhere (33). The stratified, multi-stage sampling of the Texas SPAN survey and statewide representativeness of the data is achieved by collecting representative data in each of Texas’ eight public health regions (PHRs) and by using data obtained from Texas Education Agency (TEA) on public school enrollment to create the sampling frame (weighting structure) for the study. The PHRs in Texas include: 1 (Lubbock area), 2/3 (Dallas area), 4/5 (Tyler area), 5/6 (Houston area), 7 (Austin area), 8 (San Antonio area), 9/10 (El Paso area), and 11 (Brownsville area). A comprehensive map of the Texas PHRs sampled has been previously published (33).

### Participants

The 2019–2020 Texas SPAN survey included a total of 8,710 participants in second, fourth, eighth, and 11th grades, representing a weighted sample of 1,407,016 students. The total number of fourth-grade participants, which is the sample used in the current study, was 2,897, representing a weighted sample of 355,314 fourth-grade children across Texas. The inclusion criteria for this study were the presence of completed measures of all variables of interest, which are described below in the Measures section. It is worth noting that all data were collected prior to the onset of the coronavirus disease 2019 (COVID-19) pandemic, which is why the total sample included is less than previous years, but also means estimates/results need not be interpreted through the lens of the pandemic.

### Measures

The following section details the specific measures of the Texas SPAN survey used for this study, which included PA, contexts of PA, weight status, and various demographic variables. This section also includes descriptions of how data were processed to create variables prior to analyses.

#### Physical activity

The number of days children met PA guidelines served as the main outcome of this study and was assessed by asking participants “Last week, on which days were you physically active for a total of at least 60 min per day?.” This was followed up by an explanatory sentence which stated, “Add up all the time you spent in any kind of physical activity that increased your heart rate and made you breathe hard some of the time.” Examples of physical activities were also included to aid participants’ understanding of the questions, including illustrations of activities such as basketball, soccer, running, fast dancing, swimming, tennis, and bicycling. Participants were then provided a list of each day of the week and were instructed to select all days in which they were physically active for at least 60 min that day. The number of days participants indicated they were physically active for at least 60 min per day was counted across all seven days of the week (range: 0–7) and served as the outcome variable for this study.

#### Contexts of PA

The Texas SPAN survey provides several questions related to PA context including sports participation, participation in other structured physical activities, mode of travel to school, and outdoor play. These variables were treated as the primary predictor variables for this study and are detailed below.

##### Sports participation

Participation in sports was assessed with one question which asked, “During the past 12 months, on how many sports teams did you play?,” with explicit instructions to not count physical education class. Response options included “0 teams,” “1 team,” “2 teams,” and “3 or more teams.”

##### Other organized physical activities

Participation in other organized physical activities was assessed with one question which asked, “Do you currently take part in any other organized physical activities, lessons, or classes?,” with response options of “Yes” and “No.” Examples of structured physical activities were also listed along with this question and included activities such as martial arts, dance, and gymnastics.

##### Mode of travel to school

Participants mode of travel to school was assessed by asking participants, “On most days, how do you arrive at school,” followed by several options including “walk,” “bike,” “school bus,” “city bus,” and “car.” Prior to analyses, responses were recoded to a binary ‘active travel’ variable in which walking and biking were considered active travel (1) and all other options were considered non-active travel (0).

##### Outdoor play

The number of days participants engaged in outdoor play was assessed by asking, “Last week, on which days did you play outdoors for 30 min or more?.” Participants were then provided with a list of each day of the week and were instructed to select all days on which they played outdoors. The number of days participants indicated they played outdoors was totaled across all seven days of the week and then recoded as a categorical variable with three levels: 1 = 0 days, 2 = 1–3 days, 3 = 4–7 days.

#### Weight status

Objective measures of height and weight were used to calculate body mass index (BMI) for each participant using SAS code provided by the Centers for Disease Control and Prevention (CDC) (34) and were further classified as obesity, overweight, and healthy weight, using the CDC growth charts and current recommendations (35). Prior to analyses, a binary variable was created by collapsing obesity and overweight into one category (overweight and obesity [OWOB]) and leaving healthy weight as a separate category. Methods for collecting height and weight data during the Texas SPAN survey administration have been reported elsewhere in detail (31). Briefly, height was recorded in centimeters with a stadiometer, and weight was recorded in kilograms using a calibrated top-loading scale. Height and weight measurements were taken after participants completed the written portion of the SPAN survey and were recorded directly on the questionnaire form.

#### Demographic variables

##### Sex

Participant sex was determined with a single question which asked. “Are you a boy or girl?,” followed by the response options of “Boy” and “Girl.”

##### Race/ethnicity

The race/ethnicity of participants was determined with a single question which asked, “How do you describe yourself?.” Response options included “Black or African American,” “Latino, Hispanic, or Mexican American,” “White, Caucasian, or Anglo,” “Asian,” “American Indian or Alaska Native,” and “Native Hawaiian or Other Pacific Islander.” Prior to analyses, these responses were reduced to a three-category variable, which included “African American,” “Hispanic,” and “White/Other.”

##### Urban–rural status

A three-level categorical variable for urban–rural status was determined by leveraging data from the TEA and applying it to school districts located within each of Texas’s eight administrative PHRs. The two largest school districts in each PHR were designated as “Major Urban” districts. School districts from counties with populations above 50,000 were designated as “Urban” districts, and all other school districts not categorized as Major Urban or Urban were designated as “Rural.”

##### Economic disadvantage

Data provided by the TEA were used to calculate the percentage of children whose family qualified for federal assistance programs by school. Economic disadvantage is categorized by the TEA to include qualifying for free or reduced meals under the National School Lunch and Child Nutrition Program (36, 37) and/or families with an annual income at or below the United States poverty threshold (38). Prior to analyses, a second binary variable was created by performing a median split on the percentage of children whose family qualified for federal assistance programs by school. This variable was coded as “Higher Economic Disadvantage” and “Lower Economic Disadvantage” and was used to categorize participants prior to conducting subgroup Poisson regression analyses. This variable served as the proxy for socioeconomic status.

### Statistical analysis

The complex sampling plan of the Texas SPAN survey data, which is reported in detail elsewhere (39), was accounted for using STATA’s ‘svyset’ prefix command, and missing data were not imputed. Weighted analyses used the Taylor series linearization variance estimation (40). Both the weighted and unweighted prevalence of all descriptive variables were calculated for the total sample and boys and girls separately. Descriptive statistics were compared between boys and girls using Chi-square tests for categorical variables and t-tests for continuous variables. McNemar’s test was used to compare the proportion of days PA guidelines were met between weekdays and weekend days. Weighted Poisson regression models were employed to examine the associations between PA contexts (participation in organized sports, participation in any other organized PA, active travel to school, and outdoor play) and the number of days children met PA guidelines in the past week, adjusting for sex, race/ethnicity, OWOB, urban–rural status, and economic disadvantage. Weighted Poisson regression models were chosen to account for the weighted nature of the data and the fact that the primary outcome (number of days meeting PA guidelines) is a form of count data (41). Weighted Poisson regression models were employed for (1) the total sample, (2) for boys and girls separately, (3) for higher and lower economic disadvantage separately, and (4) boys × higher economic disadvantage, boys × lower economic disadvantage, girls × higher economic disadvantage, and girls × lower economic disadvantage separately. Separate models for boys, girls, and higher/lower socioeconomic disadvantage were chosen because sex and socioeconomic status have been shown to associate with PA in children (17, 24–26, 42). All analyses had significance established at an alpha level of *p* < 0.05 and were carried out using STATA v18.0 (StataCorp LP, College Station, Texas, United States).

## Results

### Demographic characteristics and weight status

#### Total sample

All characteristics for the total sample are shown in Table 1 as unweighted counts/means and weighted percentages. Briefly, the sample of fourth-grade children (*n* = 2,897, weighted *N* = 355,314) was 9.4 ± 0.6 years of age, 50.6% male, and 51.8% Hispanic. Most children lived in either major urban (68.4%) or urban (22.4%) areas, and the average percentage of economic disadvantage by school was 70.5 ± 18.8%.

**Table 1:** Demographic characteristics and health-related behavioral variables presented as unweighted count/mean and weighted prevalence for the total sample (2019–2020 Texas SPAN).

Characteristics and behaviors	Total	
	*n* = 2,897	
	Weighted *n* = 355,314	
	Unweighted count/mean (SD)	Weighted percent
Age (years)	9.4 (0.6)	–
Race/ethnicity		
African American	457	12.2
Hispanic	1,535	51.8
White/other	905	36.2
Urban–rural status		
Major urban	934	22.4
Urban	925	68.4
Rural	1,038	9.2
Percent economically disadvantaged fourth graders (%)	70.5	–
Overweight/obesity status		
Healthy weight	1,518	54.0
Overweight/obesity	1,379	46.0
Days meeting PA guidelines		
0	344	11.2
1	337	11.7
2	277	9.6
3	367	13.8
4	443	14.3
5	407	14.4
6	216	8.3
7	467	16.7
Days meeting PA guidelines (mean)	3.6 (2.3)	
Number of sports teams participated in past 12 months		
0	1,071	35.2
1	769	28.5
2	495	17.9
3 or more	506	18.4
Participated in any other organized physical activity		
No	1,481	50.9
Yes	1,261	49.1
Commute mode to school		
Walk	130	5.2
Bike	29	1.3
School bus	661	19.8
City bus	10	0.3
Car	2000	73.6
Carpool	–	–
Days of outdoor play in the past 7 days (mean)	3.9 (2.4)	–

#### Boys and girls

Table 2 presents characteristics for boys and girls separately. While all participants were fourth graders, boys (9.5 ± 0.6 years) were slightly older than girls (9.4 ± 0.5 years). The average percentage of children attending schools with economic disadvantage differed between boys (71.3 ± 18.5%) and girls (69.7 ± 19.2%). Almost half (46.0%) of children were classified as having OWOB, which differed significantly between boys and girls such that 50.5% of boys and 41.2% of girls were classified as having OWOB.

**Table 2:** Comparison of demographic characteristics and health-related behavioral variables presented as unweighted and weighted prevalence or mean and standard deviation between boys and girls (2019–2020 Texas SPAN).

Characteristics and behaviors	Boys		Girls		*p*-value	Higher economic disadvantage		Lower economic disadvantage		*p*-value
	*n* = 1,466		*n* = 1,431			*n* = 1,427		*n* = 1,470		
	Weighted *n* = 179,803		Weighted *n* = 175,511			Weighted *n* = 175,020		Weighted *n* = 180,294		
	Unweighted count or mean	Weighted percent	Unweighted count or mean	Weighted percent		Unweighted count or mean	Weighted percent	Unweighted count or mean	Weighted percent	
Age (years)	9.5 (0.6)	–	9.4 (0.5)	–	<0.001	9.5 (0.6)	–	9.4 (0.6)	–	0.10
Race/ethnicity					0.92					<0.001
African American	237	12.0	220	12.2		205	9.7	252	13.6	
Hispanic	738	51.3	797	52.2		992	76.2	543	36.5	
White/Other	491	36.7	414	35.6		230	14.2	675	49.9	
Urban–rural status					0.97					0.01
Major urban	480	22.4	454	22.4		568	41.3	366	10.6	
Urban	443	68.3	482	68.4		493	49.1	545	80.4	
Rural	543	9.3	495	9.1		366	9.6	559	9.0	
Percent economically disadvantaged fourth graders (%)	71.3 (18.5)	–	69.7 (19.2)	–	0.02	–	–	–	–	–
Overweight/Obesity Status					<0.001					<0.001
Healthy weight	707	49.5	811	58.8		683	47.6	835	58.0	
Overweight/obesity	759	50.5	620	41.2		744	52.4	635	42.0	
Days meeting PA guidelines					0.30					0.01
0	188	11.5	156	11.0		174	12.3	170	10.6	
1	188	13.4	149	9.8		200	16.4	137	8.7	
2	128	8.5	149	10.8		129	8.4	148	10.4	
3	165	13.1	202	14.5		166	10.9	201	15.5	
4	207	14.0	236	14.7		224	13.5	219	14.9	
5	197	14.2	210	14.6		211	16.5	196	13.1	
6	95	7.2	121	9.5		87	6.7	129	9.3	
7	271	18.1	196	15.2		213	15.5	254	17.4	
Days meeting PA guidelines (mean)	3.6 (2.4)		3.6 (2.2)		0.99	3.5 (2.3)		3.7 (2.3)		0.005
Number of sports teams participated in past 12 months					<0.001					0.02
0	455	29.9	616	40.8		569	41.5	502	31.3	
1	374	27.2	395	29.9		344	25.4	425	30.4	
2	274	19.2	221	16.5		233	16.0	262	19.1	
3 or more	324	23.7	182	12.8		248	17.0	258	19.2	
Participated in any other organized physical activity					0.25					0.07
No	799	53.6	682	48.1		738	55.0	743	48.4	
Yes	569	46.4	692	51.9		599	45.0	662	51.6	
Commute mode to school					<0.01					0.66
Walk	71	5.5	59	4.8		76	6.6	54	4.3	
Bike	22	2.1	7	0.4		15	1.3	14	1.2	
School bus	344	21.8	317	17.6		314	19.2	347	20.1	
City bus	7	0.3	3	0.2		4	0.2	6	0.3	
Car	983	70.3	1,017	77.0		975	72.6	1,025	74.2	
Carpool	–	–	–	–		–	–	–	–	
Days of outdoor play in the past 7 days (mean)	3.9 (2.5)		3.9 (2.3)		0.61	3.6 (2.4)		4.2 (2.4)		<0.001

#### Higher and lower economic disadvantage

Differences in characteristics between children attending schools with higher and lower economic disadvantage are shown in Table 2. Both the racial/ethnic distribution and the urban/rural distribution differed significantly between children attending schools with higher and lower economic disadvantage. Notably, 49.9% of children attending schools with lower economic disadvantage identified as White/Other, while 14.2% attending schools with higher economic disadvantage identified as White/Other. Also, 41.3% of children attending schools with higher economic disadvantage were from Major Urban areas while 10.6% attending schools with lower economic disadvantage were from Major Urban areas. Regarding health outcomes, 52.4% of children attending schools with higher economic disadvantage had OWOB while 42.0% attending schools with lower economic disadvantage had OWOB.

### PA guidelines and PA context

#### Total sample

**Figure 1:**
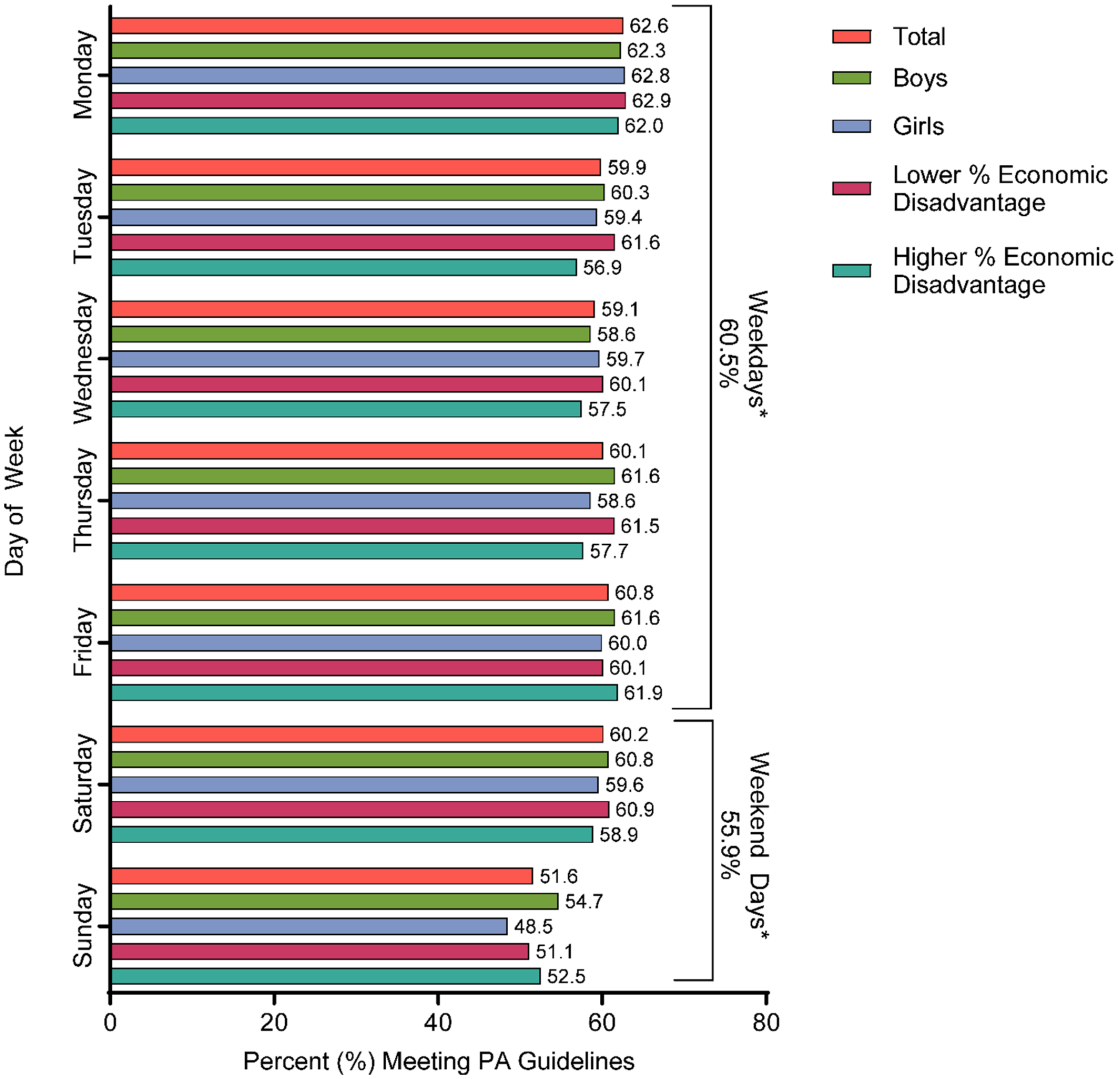
Comparison of the proportion of children meeting physical activity guidelines by weekday and weekend day. *Significant difference between the proportion of children meeting physical activity guidelines on weekdays and weekend days (McNemar’s X^2^ = 194.6, *p* < 0.001).

Daily PA guidelines were met every day of the week by 16.7% of fourth-grade children. A total of 11.2% did not meet PA guidelines on any day, while 72.1% met them between 1 and 6 days. For the total sample, the average number of days children met PA guidelines was 3.6 ± 2.3 days of the week. Figure 1 visually communicates the proportion of children meeting PA guidelines by day of the week. PA guidelines were met on 60.5% of weekdays and 55.9% of weekend days, which was a statistically significant difference. A total of 70.1% of children participated in at least one sports team in the past 12 months and 46.4% participated in other organized physical activities. Most children (70.7%) reported that a car was their typical commute mode to school, with 4.6 and 1.0% indicating they walked or rode a bike, respectively. On average, children played outdoors 3.9 ± 2.4 days of the week.

#### Boys and girls

Boys and girls differed significantly in the number of sports teams in which they participated in in the past 12 months. Compared with boys, a significantly higher percentage of girls reported participating in zero sports teams and a lower percentage of girls reported participating in three or more sports teams. The mode of travel to school also significantly differed between boys and girls. More girls reported taking a car to school compared with boys; more boys reported taking a school bus to school compared with girls; and more boys reported walking and biking to school compared with girls.

#### Higher and lower economic disadvantage

Children from schools with higher economic disadvantage met PA guidelines on fewer days compared with children from schools with lower economic disadvantage. Several PA context variables also differed significantly between children attending schools with higher and lower economic disadvantage. Compared with children from schools with lower economic disadvantage, children from schools with higher economic disadvantage participated in fewer sports teams and more children from schools with higher economic disadvantage reported participating in zero sports teams in the past 12 months. Children from schools with higher economic disadvantage also played outside on fewer days during the week compared with children from schools with lower economic disadvantage.

### Associations between meeting daily PA guidelines and PA context

#### Total sample

**Figure 2:**
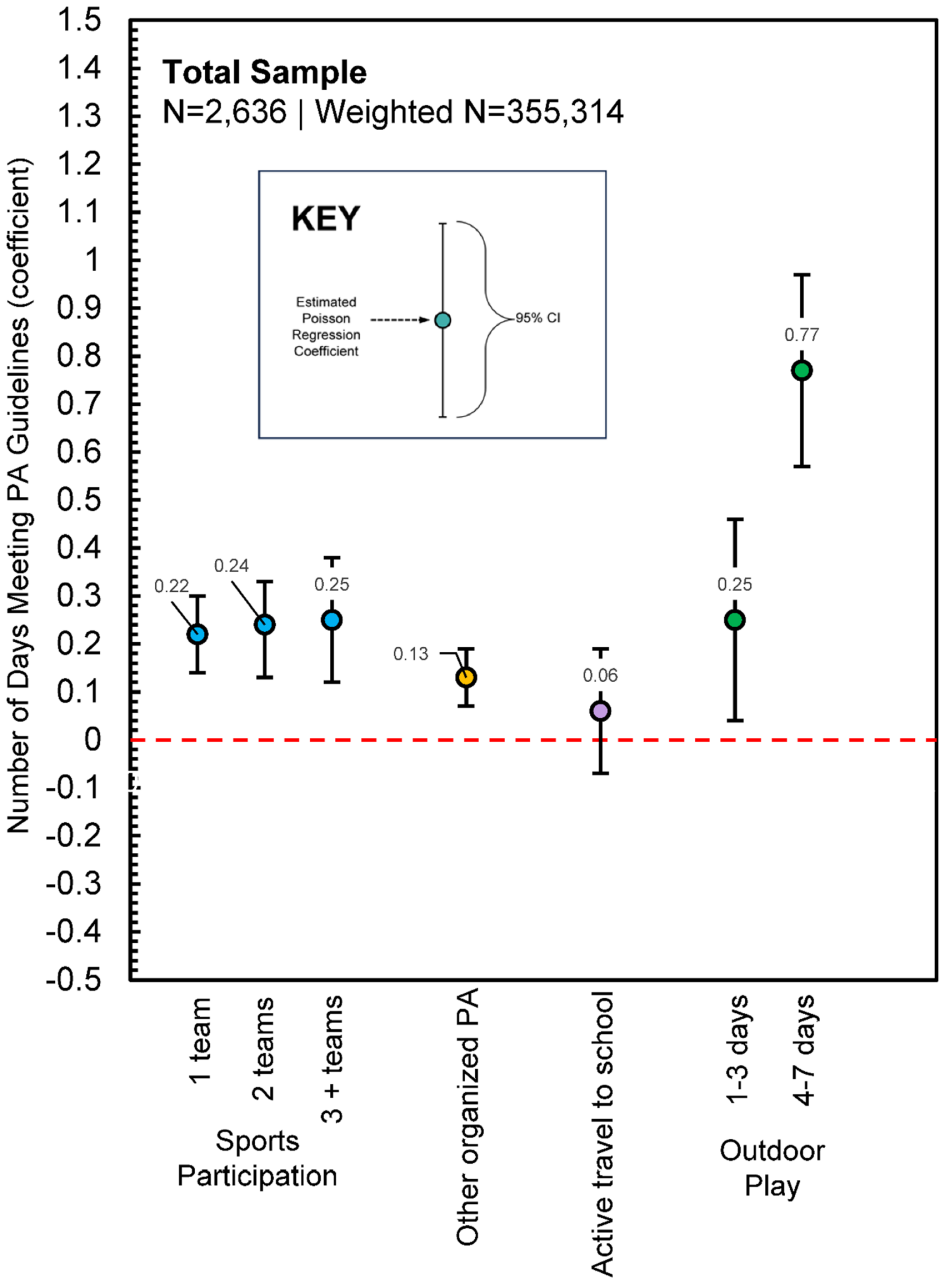
Poisson regression estimates for physical activity contexts predicting the number of days children met physical activity guidelines (Total Sample, *N* = 2,636, Weighted *N* = 355,314). Model adjusted for sex, race/ethnicity, urban/rural status, economic disadvantage, and overweight/obesity status; Supplementary Table S1 presents full model estimates.

The summary of Poisson regression analysis predicting the number of days children met daily PA guidelines for the total sample is displayed in Figure 2. Detailed estimates are shown in Supplementary Table S1. Compared to none, participating in any number of sports teams was positively associated with the number of days children met PA guidelines. Notably, a dose–response relationship was found in which participating in each additional sports team produced a stronger association with the number of days PA guidelines were met. Participation in other organized PA was also positively associated with the number of days children met PA guidelines. Playing outdoors 1–3 days and 4–7 days in the past week was positively associated with the number of days children met PA guidelines, and a dose–response relationship was found with this PA context as well. Active travel to school was not a significant predictor of meeting the PA guidelines.

#### Boys and girls

**Figure 3:**
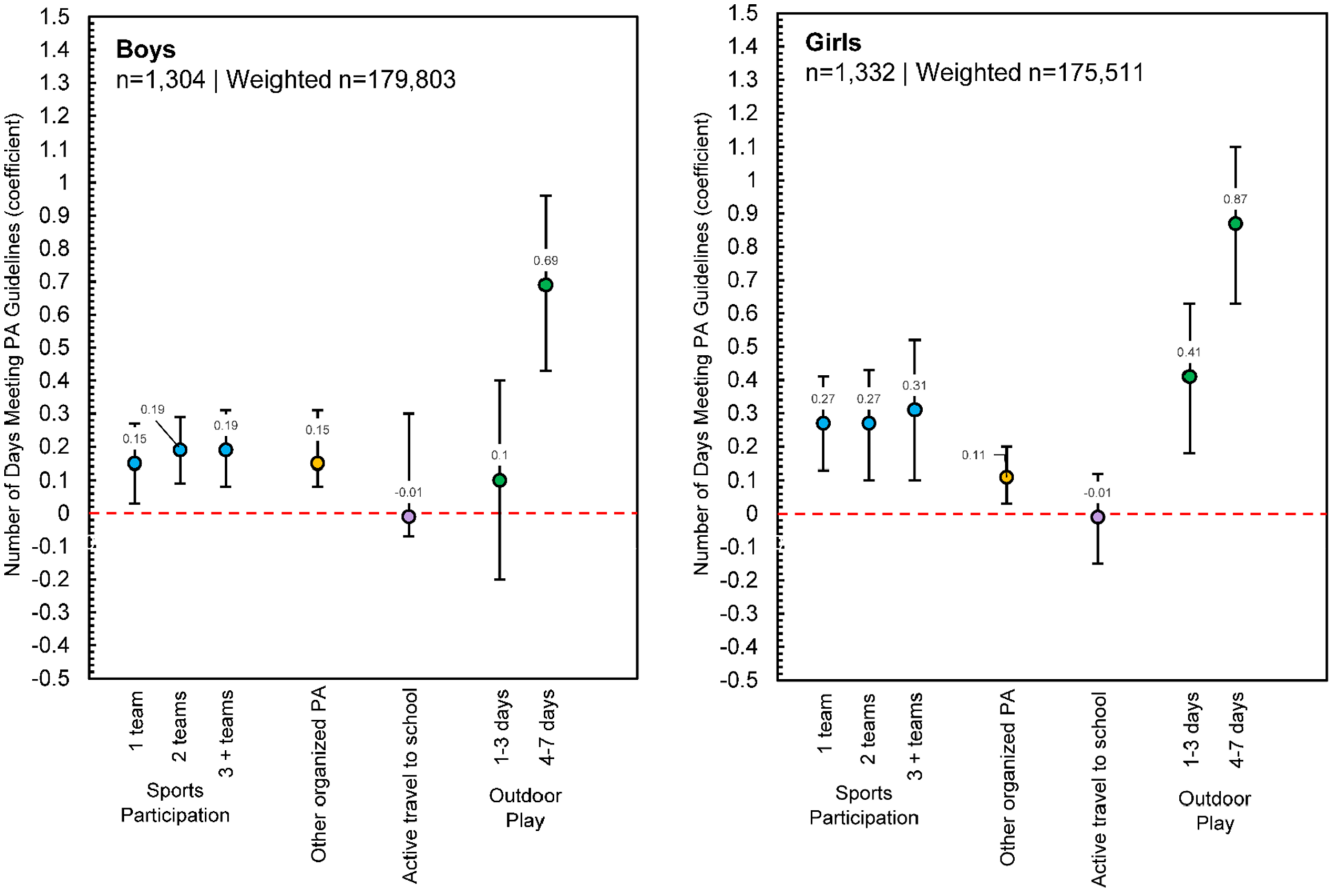
Poisson regression estimates for physical activity contexts predicting the number of days children met physical activity guidelines, reported separately for boys (*n* = 1,304, Weighted *n* = 179,803) and girls (*n* = 1,332, Weighted *n* = 175,511). *Note:* Each model adjusted for race/ethnicity, urban/rural status, economic disadvantage, and overweight/obesity status; Supplementary Table S1 presents full model estimates.

Analyses revealed some differences in both the strength and type of associations between meeting daily PA guidelines and PA context (Figure 3). Participation in sports teams was positively associated with the number of days both boys and girls met PA guidelines, but the strength of this association was higher for girls than boys. Participating in other organized PA also positively associated with the number of days boys and girls met PA guidelines. For boys, there was an apparent threshold effect, with only playing outdoors 4 or more days/week positively associated with PA guideline adherence, while for girls, playing outdoors any number of days was associated with PA guideline adherence, with a dose–response relationship noted.

#### Higher and lower economic disadvantage

**Figure 4:**
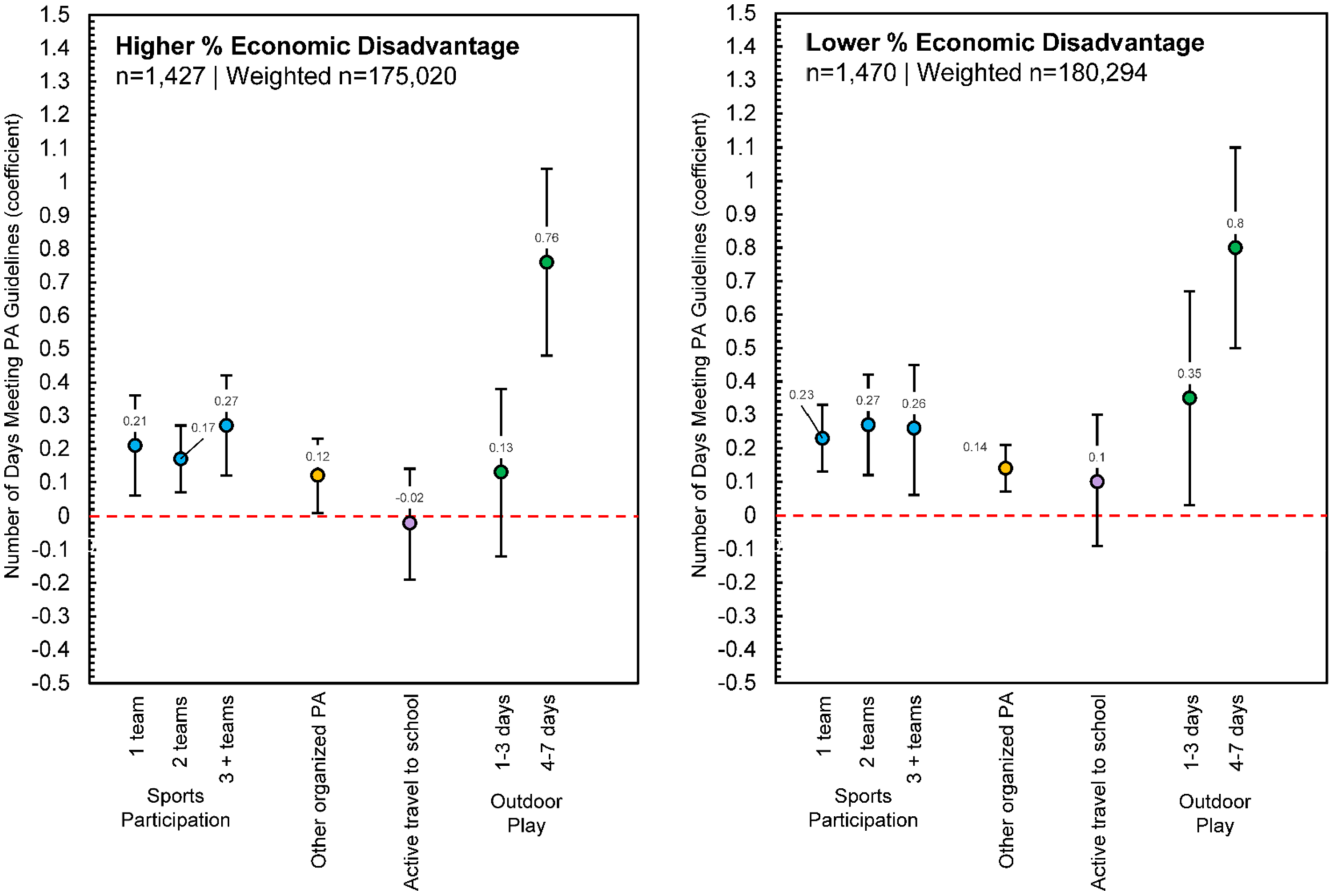
Poisson regression estimates for physical activity contexts predicting the number of days children met physical activity guidelines, reported separately for higher percent economic disadvantage (*n* = 1,427, Weighted *n* = 175,020) and lower percent economic disadvantage (*n* = 1,470, Weighted *n* = 180,294). Each model adjusted for sex, race/ethnicity, urban/rural status, and overweight/obesity status; Supplementary Table S1 presents full model estimates.

Consistent with the total sample and boys/girls, participating in sports teams positively associated with the number of days PA guidelines were met in children from schools with both higher and lower economic disadvantage, although there was not a clear dose–response relationship (Figure 4). Participation in any other organized PA also positively associated with daily PA guideline adherence for both groups. For children from schools with lower economic disadvantage, playing outdoors any number of days positively associated with daily PA guideline adherence and a clear dose–response relationship was found. For children from schools with higher economic disadvantage, only playing outdoors 4–7 days of the week was associated with PA guideline adherence.

#### Boys/girls and higher/lower economic disadvantage

**Figure 5:**
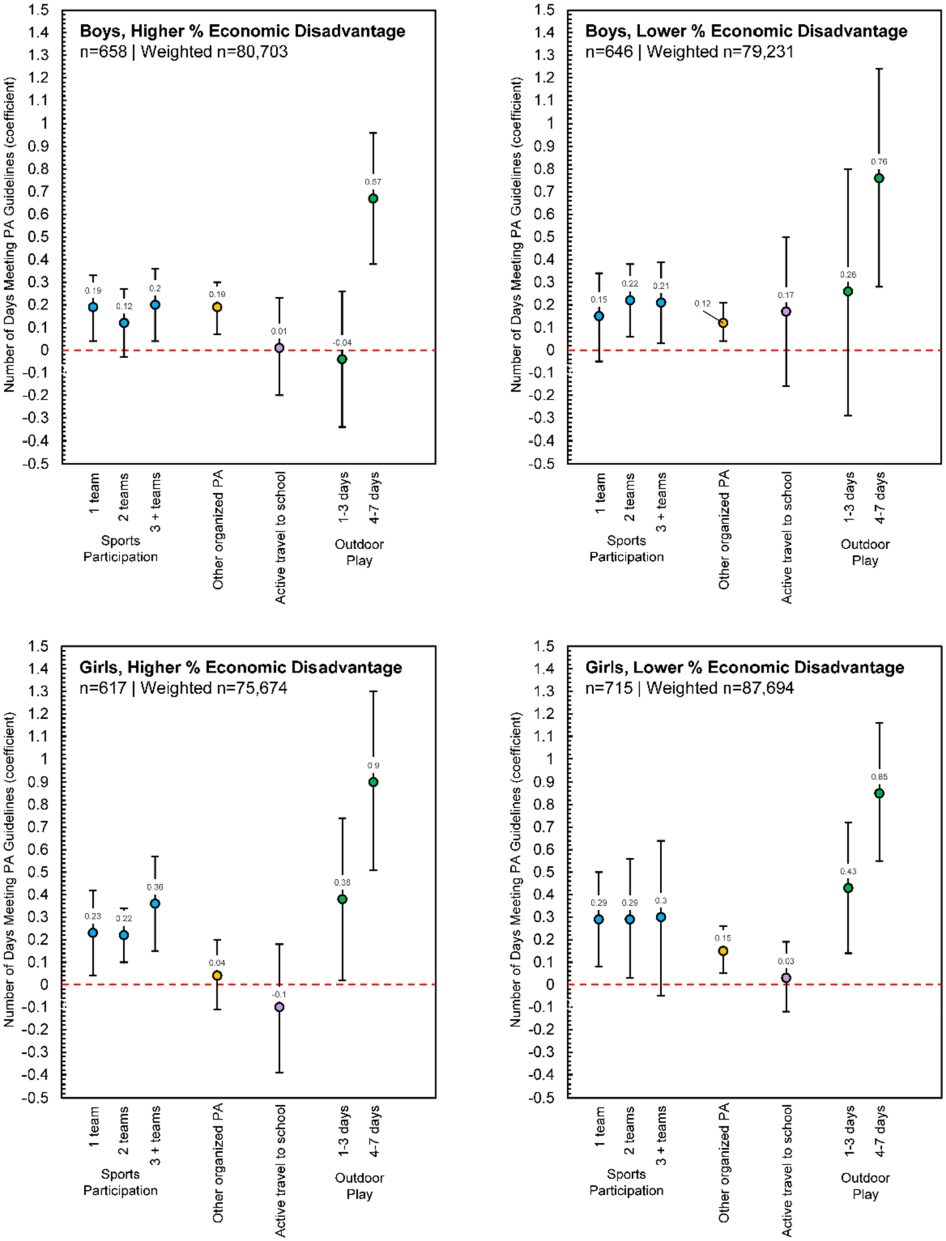
Poisson regression estimates for physical activity contexts predicting the number of days children met physical activity guidelines, reported separately for boys, higher percent economic disadvantage (*n* = 658, Weighted *n* = 80,703), boys, lower percent economic disadvantage (*n* = 646, Weighted *n* = 79,231), girls, higher percent economic disadvantage (*n* = 617, Weighted *n* = 75,674), and girls, lower percent economic disadvantage (*n* = 715, Weighted *n* = 87,694). Each model adjusted for race/ethnicity, urban/rural status, and overweight/obesity status; Supplementary Table S2 presents full model estimates.

Some subgroup variability in estimates was evident among the analyses stratified by sex (boys/girls) and higher/lower economic disadvantage (Figure 5). Participating in sports teams positively associated with the number of days PA guidelines were met for each group, but the significant association only held constant for each additional sports team for girls from schools with higher economic disadvantage. The dose–response relationship was most notable for this group as well. Conversely, girls from schools with higher economic disadvantage were the only group in which participation in any other organized PA was not a significant predictor of daily PA guideline adherence. Finally, while the dose–response relationship for days of outdoor play was consistent across girls from schools with both high and low economic disadvantage, only 4–7 days of outdoor play was a significant predictor of daily PA guideline adherence for boys from schools with both high and low economic disadvantage.

## Discussion

This study was a cross-sectional examination of daily PA guideline adherence in relation to several PA contexts using data from the 2019–2020 Texas SPAN survey. We sought to examine associations between PA contexts (sports participation and other out-of-school structured physical activities, active travel to school, and outdoor play), and the number of days fourth-grade children met PA guidelines in a representative sample of children living in Texas. We were also interested in comparing PA guideline adherence and PA context between boys/girls and between participants from schools with higher/lower levels of economic disadvantage. Overall, we found a significant dose–response relationship between sports participation and the number of days fourth-grade children met PA guidelines. We also found a similar dose–response relationship between the number of days children played outside and the number of days children met PA guidelines. Participating in other organized PA was also positively associated with the number of days children met PA guidelines but active travel to school was not. Several differences between boys/girls and children from schools with higher/lower levels of economic disadvantage were noted, but playing outdoors was a consistent predictor of PA guideline adherence across all model comparisons in this age group. Results shed light on how different PA contexts may associate with PA guideline adherence and identify potential salient intervention components for those designing and conducting PA-based health behavior interventions for children. Comparisons between boys/girls and children from lower/higher economic disadvantage also emphasize the need for more equitable PA promotion strategies focused on girls and on children attending schools with higher economic disadvantage.

The average number of days per week fourth-grade children living in Texas met PA guidelines (3.6 ± 2.3 days) is difficult to compare with much of the previously published literature, as most studies simply report the proportion of children meeting PA guidelines. A previous study using accelerometer-derived data reported the proportion of days fourth-grade children met PA guidelines was 47.5% (43), which is similar to our study ([3.6/7]*100% = 51.4%). Comparing the proportion of children meeting PA guidelines proves difficult, as many studies derive this proportion by averaging PA across all measured days, which does not consider the “daily” aspect of the guideline language (5–8). For our study, we accounted for each individual day of the week and found about 15% of children met daily PA guidelines, meaning 15% of children met the PA guideline every single day of the week. Other studies from the US have reported 23% (44), 71% (45), and even 91.5% (46) of youth meet PA guidelines, but again these estimates are difficult to compare due to differences in data handling strategies (47). For studies outside of the US that have operationalized the “daily” aspect of PA guideline adherence, estimates are comparable (42).

We also found children from schools with higher economic disadvantage met PA guidelines on fewer days than children from schools with lower economic disadvantage and this finding aligns with previously published literature involving samples from both inside and outside of the US that has explored various proxies for socioeconomic status and PA (24, 25, 48–50). There is no single reason for this disparity, but some include both financial and environmental accessibility to facilities and organized sports/activities that promote PA, neighborhood safety which may limit the ability to play outside or walk/bike to school, and the moderating effect of weight status, which has shown to favor children with higher socioeconomic status (26). In Texas, the percentage of children who experience socioeconomic disadvantage is higher than many other states, with 38% of families falling 400% below the federal poverty level, 19.6% of children experiencing poverty, and the median household income being $3,000 less than national average (51). This is also evidenced by the relatively high median of children from schools with higher economic disadvantage in our sample.

The results of our study also indicate children from schools with higher economic disadvantage participate in fewer sports teams. In terms of PA context, sports participation may be the most cost prohibitive. In fact, the average cost of participating in a single sport has been estimated to be $883 for children in the United States, with some families spending upward of $4,000 annually (19), making it an opportunity not afforded by every child. When making comparisons between boys and girls, we found girls participated in fewer sports teams as well. This is concerning as sports can have a profound impact on not only PA (16), and more specifically, MVPA (15), but children may also experience other benefits from sports participation including improved mental health (52), decreased risk of cardiovascular disease (53) and overweight/obesity (54), and higher academic achievement (55).

Regarding some of the other PA contexts we explored, our results indicate children from schools with higher economic disadvantage played outside on fewer days than children from schools with lower economic disadvantage. While playing outdoors is arguably less expensive than participating in sports, there still may be costs associated with playing outdoors, albeit in a more indirect manner (21–23, 56). Still, children living in neighborhoods with higher socioeconomic disadvantage have been shown to have better accessibility to opportunities for outdoor play, although this is variable by country and region (18, 57). However, neighborhoods with higher socioeconomic disadvantage tend to be less safe (18), limiting the chances that children will utilize these opportunities. Children from families with lower socioeconomic status also tend to face more restrictions on outdoor play than those from households with higher economic status, with evidence suggesting safety is a significant concern (58). Outdoor play can also occur around home environments but it has been found that families with lower socioeconomic status provide more opportunities for sedentary behavior and fewer opportunities to be physically active (58), which may also be driving these disparities.

While we did not find differences in commute mode to school when comparing children from schools with higher and lower economic disadvantage as others have (59, 60), we did find differences in commute mode to school between boys and girls, such that more boys indicated walking, biking, and taking a school bus. Perceived safety and the level of independent mobility given to boys and girls may contribute to this finding (61–66), although we did not assess these variables in this study. Programs, policies, and/or interventions that aim to promote active commuting to school should consider these sex-based differences and offer tailored strategies to alleviate parental concerns. In general, we found that a very small percentage of fourth-grade children in Texas reported walking and biking to school (6.5% overall), and this small percentage of active commuting is reflected in other state-level and national estimates (60, 67–69). This is a concerning statistic, especially since as children get older this percentage tends to decline past sixth grade (70). Regardless of sex or socioeconomic status, more effort should be put into promoting active travel among children by addressing concerns around safety, improving built environment infrastructure supporting walking and biking, and increasing promotion efforts.

In terms of the strength of associations between the number of days children met PA guidelines and different PA contexts, several important findings were noted. First, for the total sample, for girls, and for girls from schools with higher economic disadvantage, there was a clear dose–response relationship between the number of sports teams in which they participated and the number of days PA guidelines were met. This dose–response relationship was observed for other groups as well, but not in as consistent of a manner. Sports have long been an important intervention component for youth PA promotion, and there are several examples of these types of interventions in the literature (71). Findings from our study create an impetus for more sports-based interventions specifically designed for girls and for girls from schools with higher economic disadvantage as we found (1) girls and children from schools with higher economic disadvantage participated in fewer sports than boys and children from schools with lower economic disadvantage and (2) the association between sports participation and the number of days PA guidelines were met was stronger for girls and girls from schools with higher economic disadvantage than boys and children from lower economic disadvantage. There are few examples of sports-based interventions specifically for girls (72–74), but there have been several observational studies on various aspects of girls’ sports participation (27, 75–80). More work should be put into these efforts and in attempting to scale programs up to increase reach to girls and children from schools with higher economic disadvantage.

Another important finding was the strength of the association between the number of days PA guidelines were met and outdoor play. Not only was there a clear dose–response relationship for many of the groups, but the strength of the association for 4–7 days of outdoor play was markedly higher than all other PA contexts. In many models, the strength of the association was 2–3 times higher than participating in three or more sports teams throughout the year. Recent work, sometimes categorized as and/or having overlap with “risky play” (81, 82), “unstructured play” (83), “nature play” (84), and/or “free play” (85), highlights the uniqueness of this PA context and offers an exciting avenue for youth PA promotion (20). Lee et al. (83) conducted a review of the correlates of outdoor play among children and found that individual, parental, home, and social environments influence the time spent playing outdoors. Based on this review, factors such as independent mobility, overweight status, parents’ attitudes, concerns, and behavior, peer influence, housing type, and, supporting our findings, proxies for socioeconomic status, all play a role in influencing the amount of time children spend playing outdoors. Intervention efforts have yielded promising results for the efficacy of outdoor play increasing PA among children as well (86). It is worth noting many outdoor play studies and interventions have been conducted with younger children (preschool and Kindergarten), while not as much attention has been given to older children and adolescents. As this area of research grows, researchers should consider expanding investigations to older children and should also explore how outdoor play may track into adolescence and even adulthood, as parenting practices have been shown to influence outdoor play in children as well (23).

Finally, we found other organized PA positively associated with the number of days PA guidelines were met across most groups, except for boys from schools with lower economic disadvantage and girls from schools with higher economic disadvantage. While the survey question we used did not ask about specifics, the fact that any other organized PA positively associated with PA guideline adherence provides further support for the structured days hypothesis (SDH) (87), which posits obesity-related behaviors in children may be beneficially regulated by formal structure, in this case, organized PA outside of the school context. As with sports, participation in other organized PA can come with a financial burden, although we did not find significant differences in other organized PA participation between children from schools with higher and lower economic disadvantage. Using a more immediate proxy for children’s socioeconomic status may have revealed significant differences, as previous literature has shown (88, 89), but we cannot be certain that is the case in our sample. Still, other organized PA may be a viable PA promotion alternative to sports, especially for children who are not interested in traditional sports, if economic barriers are addressed. Indeed, almost half of the fourth-grade children from our sample indicated participating in other organized PA throughout the year, and this estimate was not much different for children from schools with higher economic disadvantage. We also found that PA guidelines were met more frequently on weekdays compared with weekend days, which lends further support to the SDH, as weekend days tend to be less structured (87). Much like the summer months, PA intervention efforts should focus on providing opportunities for children on weekend days, which lack the formal structure school days provide during the week.

### Strengths and limitations

The Texas SPAN survey provides a unique opportunity to leverage data that are representative of the entire state of Texas, which happens to be the second most populous state in the US (90) and is home to 7.5 million children, accounting for 10% of all children in the US (30). Participants in the Texas SPAN survey reflect the racial/ethnic and economic diversity of the state as well. Because of the questions asked in the SPAN survey, we were also able to conduct one of the first studies exploring how several different PA contexts associate with PA guideline adherence in a representative sample of fourth-grade children. As previously highlighted, this approach allows us to compare PA behaviors across contexts within the same sample of participants in a naturalistic setting, as past PA interventions have typically not compared how PA contexts may differentially impact PA outcomes across several domains (sports, outdoor play, structured activities, etc.) Results may be valuable to those wishing to conduct further research utilizing device-based measures of PA and for researchers hoping to design effective PA-based interventions for children of a similar age. However, results should also be interpreted with study limitations in mind. A clear limitation is the self-reported and cross-sectional nature of our study design, which limits us to only interpreting associations between PA context and PA guideline adherence and barring us from making any causal interpretations with the data. Another limitation is our inability to account for several school-based PA contexts, including recess and physical education. Survey questions regarding structured PA did make it clear not to include physical education classes in participant responses, but having information on recess and physical education would enrich the analyses. Finally, temporal differences in how certain questions were worded in the survey should be acknowledged. For example, children were asked to indicate how many sports teams they were on “in the past 12 months,” how they traveled to school “on most days,” and how many days they played outside “in the past week.” These differences in temporality may have influenced the way in which questions were interpreted, answers were provided, and subsequent interpretations of the associations between these contexts and the number of days PA guidelines were met. For example, the self-reported “dose” of physical activity for questions that had participants report the frequency of participating in a structured activity “in the past week” could have potentially been higher than questions that had participants report the frequency of participating in a structured activity “in the past 12 months” and/or “on most days,” which could result in stronger or weaker associations between certain types of structured activities and meeting PA guidelines.

## Conclusion

Participating in organized sports and other structured physical activities, in addition to playing outdoors, may beneficially influence the number of days fourth-grade children meet PA guidelines, although there are sex- and economic-based disparities present. Programs that aim to enhance PA in children should consider these contextual factors in light of these disparities and further investigate how to promote sports, organized activities, and outdoor play effectively and appropriately among children, especially for girls and for children from schools with higher economic disadvantage. With results being generalizable to only fourth-grade children in Texas, USA, future work should be continued in other countries and cultures to investigate how certain contexts might differentially influence PA guideline adherence. Because participation in certain PA contexts may decline as children get older [e.g., walking/biking to school (70) and types of outdoor play (91)], more research and health promotion work should be conducted with adolescent participants to see if the relationships found in our study are maintained as age increases. Future studies should also employ more rigorous observational investigations with device-based measures of PA and should collect day-level contextual information about PA opportunities and their utilization. Future studies should also explore how PA contexts influence PA across the lifespan and how the context of PA might change as children get older. Our study highlights there is not a “one size fits all” approach to PA promotion for children. Sex- and economic-based differences in participation in different PA contexts and differences in the strength of associations between PA context and PA guideline adherence underscore what may be viable for some children may not be for other children, and interventions and programs hoping to promote PA in children should respond appropriately.

## Data availability statement

The full datasets presented in this article are not readily available because public access was not specified in the consent forms. Reasonable requests to access the datasets should be directed to Deanna.M.Hoelscher@uth.tmc.edu. Limited datasets can be accessed at https://span-interactive.sph.uth.edu/.

## Ethics statement

The studies involving humans were approved by the Committee for the Protection of Human Subjects at the University of Texas Health Science Center at Houston (UTHealth Houston) (HSC-SPH-18-0432), the Texas Department of State Health Services Institutional Review Board, and local school district review committees. The studies were conducted in accordance with the local legislation and institutional requirements. Written informed consent for participation in this study was provided by the participants’ legal guardians/next of kin.

## Author contributions

CP: Conceptualization, Formal analysis, Investigation, Methodology, Software, Visualization, Writing – original draft, Writing – review & editing. DB: Conceptualization, Investigation, Methodology, Writing – original draft, Writing – review & editing. NR: Conceptualization, Data curation, Formal analysis, Methodology, Writing – original draft, Writing – review & editing. AS: Conceptualization, Writing – original draft, Writing – review & editing. RM: Data curation, Formal analysis, Methodology, Project administration, Resources, Writing – original draft, Writing – review & editing. DS: Conceptualization, Methodology, Writing – original draft, Writing – review & editing. DH: Conceptualization, Data curation, Funding acquisition, Investigation, Methodology, Supervision, Writing – original draft, Writing – review & editing.
