# Supplementary material for: Examining associations between physical activity context and children meeting daily physical activity guidelines: the role of outdoor play, sports, and other organized activities
Source: Front Public Health. 2024 May 20;12:1352644. doi: 10.3389/fpubh.2024.1352644 (PMC11144898; doi:10.3389/fpubh.2024.1352644)
Supplement: Supplementary file 1 [file Data_Sheet_1.docx]

**Supplementary Table 1.** Summary of Poisson regression analysis predicting the number of days 4^th^ grade children met physical activity guidelines for the total sample, boys/girls, and low percent/high percent economic disadvantage, separately (2019-2020 Texas SPAN).

| **Predictor** | **Total Sample**  n=2,636  Weighted n=355,314 | | **Boys**  n=1,304  Weighted n=179,803 | | **Girls**  n=1,332  Weighted n=175,511 | | **Lower % Econ. Dis.**  n=1,361  Weighted n=175,020 | | **Higher % Econ. Dis.** n=1,275  Weighted n=180,294 | |
| --- | --- | --- | --- | --- | --- | --- | --- | --- | --- | --- |
|  | b-coefficient | 95%CI | b-coefficient | 95%CI | b-coefficient | 95%CI | b-coefficient | 95%CI | b-coefficient | 95%CI |
| Number of sports teams participated in past 12 months (none as referent) |  |  |  |  |  |  |  |  |  |  |
| 1 | **0.22** | 0.14, 0.30 | **0.15** | 0.03, 0.27 | **0.27** | 0.13, 0.41 | **0.23** | 0.13, 0.33 | **0.21** | 0.06, 0.36 |
| 2 | **0.24** | 0.13, 0.33 | **0.19** | 0.09, 0.29 | **0.27** | 0.10, 0.43 | **0.27** | 0.12, 0.42 | **0.17** | 0.07, 0.27 |
| 3 or more | **0.25** | 0.12, 0.38 | **0.19** | 0.08, 0.31 | **0.31** | 0.10, 0.52 | **0.26** | 0.06, 0.45 | **0.27** | 0.12, 0.42 |
| Participated in any other organized physical activity (No as referent) | **0.13** | 0.07, 0.19 | **0.15** | 0.08, 0.31 | **0.11** | 0.03, 0.20 | **0.14** | 0.07, 0.21 | **0.12** | 0.01, 0.23 |
| Active travel to school (Passive travel referent) | 0.06 | -0.07, 0.19 | 0.11 | -0.07, 0.30 | -0.01 | -0.15, 0.12 | 0.10 | -0.09, 0.30 | -0.02 | -0.19, 0.14 |
| Days of outdoor play in the past 7 days (0 days as referent) |  |  |  |  |  |  |  |  |  |  |
| 1-3 days | **0.25** | 0.04, 0.46 | 0.10 | -0.20, 0.40 | **0.41** | 0.18, 0.63 | **0.35** | 0.03, 0.67 | 0.13 | -0.12, 0.38 |
| 4-7 days | **0.77** | 0.57, 0.97 | **0.69** | 0.43, 0.96 | **0.87** | 0.63, 1.10 | **0.80** | 0.50, 1.10 | **0.76** | 0.48, 1.04 |
| Sex (Female referent) | -0.02 | -0.10, 0.05 | - |  | - |  | -0.03 | -0.13, 0.07 | 0.01 | -0.08, 0.09 |
| Race/Ethnicity (Hispanic referent) |  |  |  |  |  |  |  |  |  |  |
| African American | **-0.10** | -0.17, -0.03 | **-0.14** | -0.27, -0.01 | -0.07 | -0.17, 0.03 | **-0.16** | -0.24, -0.07 | 0.04 | -0.07, 0.15 |
| White/Other | 0.02 | -0.04, 0.07 | 0.01 | -0.09, 0.11 | 0.01 | -0.08, 0.10 | 0.04 | -0.02, 0.09 | -0.05 | -0.21, 0.11 |
| Urbanicity (Major Urban referent) |  |  |  |  |  |  |  |  |  |  |
| Urban | **-0.07** | -0.13, -0.01 | -0.09 | -0.18, 0.01 | -0.06 | -0.16, 0.03 | -0.06 | -0.14, 0.02 | -0.04 | -0.13, 0.05 |
| Rural | -0.02 | -0.09, 0.05 | -0.01 | -0.09, 0.08 | -0.03 | -0.12, 0.04 | 0.03 | -0.06, 0.11 | -0.07 | -0.17, 0.03 |
| Percent economically disadvantaged 4^th^ graders | 0.01 | -0.13, 0.16 | 0.02 | -0.21, 0.25 | -0.01 | -0.27, 0.26 | - | - | - | - |
| Overweight/Obesity status (Healthy weight referent) | **-0.08** | -0.13, -0.02 | -0.08 | -0.18, 0.01 | **-0.08** | -0.14, -0.01 | **-0.08** | -0.15, -0.01 | -0.07 | -0.15, 0.01 |

**Supplementary Table 2.** Summary of Poisson regression analysis predicting the number of days 4^th^ grade children met physical activity guidelines for combinations of boys/girls and low percent/high percent economic disadvantage (2019-2020 Texas SPAN).

| **Predictor** | **Girls, Low % Econ. Dis.**  n=715  Weighted n=87,694 | | **Girls, High % Econ. Dis.**  n=617  Weighted n=75,674 | | **Boys, Low % Econ. Dis.**  n=646  Weighted n=79,231 | | **Boys, High % Econ. Dis.**  n=658  Weighted n=80,703 | |
| --- | --- | --- | --- | --- | --- | --- | --- | --- |
|  | b-coefficient | 95%CI | b-coefficient | 95%CI | b-coefficient | 95%CI | b-coefficient | 95%CI |
| Number of sports teams participated in past 12 months (none as referent) |  |  |  |  |  |  |  |  |
| 1 | **0.29** | 0.08, 0.50 | **0.23** | 0.04, 0.42 | 0.15 | -0.05, 0.34 | **0.19** | 0.04, 0.33 |
| 2 | **0.29** | 0.03, 0.56 | **0.22** | 0.10, 0.34 | **0.22** | 0.06, 0.38 | 0.12 | -0.03, 0.27 |
| 3 or more | 0.30 | -0.05, 0.64 | **0.36** | 0.15, 0.57 | **0.21** | 0.03, 0.39 | **0.20** | 0.04, 0.36 |
| Participated in any other organized physical activity (No as referent) | **0.15** | 0.05, 0.26 | 0.04 | -0.11, 0.20 | **0.12** | 0.04, 0.21 | **0.19** | 0.07, 0.30 |
| Active travel to school (Passive travel referent) | 0.03 | -0.12, 0.19 | -0.10 | -0.39, 0.18 | 0.17 | -0.16, 0.50 | 0.01 | -0.20, 0.23 |
| Days of outdoor play in the past 7 days (0 days as referent) |  |  |  |  |  |  |  |  |
| 1-3 days | **0.43** | 0.14, 0.72 | **0.38** | 0.02, 0.74 | 0.26 | -0.29, 0.80 | -0.04 | -0.34, 0.26 |
| 4-7 days | **0.85** | 0.55, 1.16 | **0.90** | 0.51, 1.30 | **0.76** | 0.28, 1.24 | **0.67** | 0.38, 0.96 |
| Race/Ethnicity (Hispanic referent) |  |  |  |  |  |  |  |  |
| African American | -0.11 | -0.25, 0.03 | 0.06 | -0.04, 0.17 | -0.20 | -0.41, 0.01 | 0.03 | -0.12, 0.19 |
| White/Other | -0.03 | -0.06, 0.12 | -0.04 | -0.28, 0.19 | 0.03 | -0.07, 0.14 | -0.06 | -0.27, 0.14 |
| Urbanicity (Major Urban referent) |  |  |  |  |  |  |  |  |
| Urban | -0.04 | -0.19, 0.11 | -0.05 | -0.14, 0.04 | -0.08 | -0.17, 0.01 | -0.05 | -0.20, 0.11 |
| Rural | 0.01 | -0.14, 0.15 | -0.06 | -0.17, 0.05 | 0.06 | -0.02, 0.15 | -0.07 | -0.20, 0.07 |
| Overweight/Obesity status (Healthy weight referent) | -0.08 | -0.18, 0.02 | -0.07 | -0.17, 0.02 | -0.08 | -0.23, 0.06 | -0.07 | -0.17, 0.04 |
